# Group work as an incentive for learning – students’ experiences of group work

**DOI:** 10.3389/fpsyg.2014.00558

**Published:** 2014-06-05

**Authors:** Eva Hammar Chiriac

**Affiliations:** Division of Psychology, Department of Behavioural Sciences and Learning, Linköping UniversityLinköping, Sweden

**Keywords:** group work, collaborative learning, cooperative learning, higher education, students’ perspectives, qualitative research

## Abstract

Group work is used as a means for learning at all levels in educational systems. There is strong scientific support for the benefits of having students learning and working in groups. Nevertheless, studies about what occurs in groups during group work and which factors actually influence the students’ ability to learn is still lacking. Similarly, the question of why some group work is successful and other group work results in the opposite is still unsolved. The aim of this article is to add to the current level of knowledge and understandings regarding the essence behind successful group work in higher education. This research is focused on the students’ experiences of group work and learning in groups, which is an almost non-existing aspect of research on group work prior to the beginning of the 21st century. A primary aim is to give university students a voice in the matter by elucidating the students’ positive and negative points of view and how the students assess learning when working in groups. Furthermore, the students’ explanations of why some group work ends up being a positive experience resulting in successful learning, while in other cases, the result is the reverse, are of interest. Data were collected through a study-specific questionnaire, with multiple choice and open-ended questions. The questionnaires were distributed to students in different study programs at two universities in Sweden. The present result is based on a reanalysis and qualitative analysis formed a key part of the study. The results indicate that most of the students’ experiences involved group work that facilitated learning, especially in the area of academic knowledge. Three important prerequisites (learning, study-social function, and organization) for group work that served as an effective pedagogy and as an incentive for learning were identified and discussed. All three abstractions facilitate or hamper students’ learning, as well as impact their experiences with group work.

## INTRODUCTION

Group work is used as a means for learning at all levels in most educational systems, from compulsory education to higher education. The overarching purpose of group work in educational practice is to serve as an incentive for learning. For example, it is believed that the students involved in the group activity should “learn something.” This prerequisite has influenced previous research to predominantly focus on how to increase efficiency in group work and how to understand why some group work turns out favorably and other group work sessions result in the opposite. The review of previous research shows that in the 20th century, there has been an increase in research about students’ cooperation in the classroom (Lou et al., 1996; Gillies and Boyle, 2010, 2011). This increasing interest can be traced back to the fact that both researchers and teachers have become aware of the positive effects that collaboration might have on students’ ability to learn. The main concern in the research area has been on how interaction and cooperation among students influence learning and problem solving in groups (Hammar Chiriac, 2011a,b).

Two approaches concerning learning in group are of interest, namely *cooperative learning* and *collaborative learning*. There seems to be a certain amount of confusion concerning how these concepts are to be interpreted and used, as well as what they actually signify. Often the conceptions are used synonymously even though there are some differentiations. Cooperative group work is usually considered as a comprehensive umbrella concept for several modes of student active working modes (Johnson and Johnson, 1975; Webb and Palincsar, 1996), whereas *collaboration* is a more of an exclusive concept and may be included in the much wider concept cooperation (Hammar Chiriac, 2011a,b). Cooperative learning may describe group work without any interaction between the students (i.e., the student may just be sitting next to each other; Bennet and Dunne, 1992; Galton and Williamson, 1992), while collaborative learning always includes interaction, collaboration, and utilization of the group’s competences (Bennet and Dunne, 1992; Galton and Williamson, 1992; Webb and Palincsar, 1996).

At the present time, there is strong scientific support for the benefits of students learning and working in groups. In addition, the research shows that collaborative work promotes both academic achievement and collaborative abilities (Johnson and Johnson, 2004; Baines et al., 2007; Gillies and Boyle, 2010, 2011). According to Gillies and Boyle (2011), the benefits are consistent irrespective of age (pre-school to college) and/or curriculum. When working interactively with others, students learn to inquire, share ideas, clarify differences, problem-solve, and construct new understandings. Gillies (2003a,b) also stresses that students working together are more motivated to achieve than they would be when working individually. Thus, group work might serve as an incentive for learning, in terms of both academic knowledge and interpersonal skills. Nevertheless, studies about what occur in groups during group work and which factors actually influence the students’ ability to learn is still lacking in the literature, especially when it comes to addressing the students’ points of view, with some exceptions (Cantwell and Andrews, 2002; Underwood, 2003; Peterson and Miller, 2004; Hansen, 2006; Hammar Chiriac and Granström, 2012). Similarly, the question of why some group work turns out successfully and other work results in the opposite is still unsolved. In this article, we hope to contribute some new pieces of information concerning the why some group work results in positive experiences and learning, while others result in the opposite.

## GROUP WORK IN EDUCATION

Group work is frequently used in higher education as a pedagogical mode in the classroom, and it is viewed as equivalent to any other pedagogical practice (i.e., whole class lesson or individual work). Without considering the pros and cons of group work, a non-reflective choice of pedagogical mode might end up resulting in less desirable consequences. A reflective choice, on the other hand, might result in positive experiences and enhanced learning (Galton et al., 2009; Gillies and Boyle, 2011; Hammar Chiriac and Granström, 2012).

## GROUP WORK AS OBJECTIVE OR MEANS

Group work might serve different purposes. As mentioned above, the overall purpose of the group work in education is that the students who participate in group work “learn something.” Learning can be in terms of academic knowledge or “group knowledge.” Group knowledge refers to learning to work in groups (Kutnick and Beredondini, 2009; Gillies and Boyle, 2010, 2011; Hammar Chiriac, 2011a,b). Affiliation, fellowship, and welfare might be of equal importance as academic knowledge, or they may even be prerequisites for learning. Thus, the group and the group work serve more functions than just than “just” being a pedagogical mode. Hence, before group work is implemented, it is important to consider the purpose the group assignment will have as the objective, the means, or both.

From a learning perspective, group work might function as both *an objective* (i.e., learning collaborative abilities) and *as the means* (i.e., a base for academic achievement) or* both* (Gillies, 2003a,b; Johnson and Johnson, 2004; Baines et al., 2007). If the purpose of the group work is to serve as an objective, the group’s function is to promote students’ development of group work abilities, such as social training and interpersonal skills. If, on the other hand, group work is used as a means to acquire academic knowledge, the group and the collaboration in the group become a base for students’ knowledge acquisition (Gillies, 2003a,b; Johnson and Johnson, 2004; Baines et al., 2007). The group contributes to the acquisition of knowledge and stimulates learning, thus promoting academic performance. Naturally, group work can be considered to be a learning environment, where group work is used both as an objective and as the means. One example of this concept is in the case of tutorial groups in problem-based learning. Both functions are important and might complement and/or even promote each other. Albeit used for different purposes, both approaches might serve as an incentive for learning, emphasizing different aspect knowledge, and learning in a group within an educational setting.

### WORKING IN A GROUP OR AS A GROUP

Even if group work is often defined as “pupils working together as a group or a team,” (Blatchford et al., 2003, p. 155), it is important to bear in mind that group work is not just one activity, but several activities with different conditions (Hammar Chiriac, 2008, 2010). This implies that group work may change characteristics several times during a group work session and/or during a group’s lifetime, thus suggesting that certain working modes may be better suited for different parts of a group’s work and vice versa (Hammar Chiriac, 2008, 2010). It is also important to differentiate between how the work is accomplished in the group, whether by working in a group or working as a group.

From a group work perspective, there are two primary ways of discussing cooperation in groups: working *in a group* (cooperation) or working *as a group* (collaboration; Underwood, 2003; Hammar Chiriac and Granström, 2012). Situations where students are sitting together in a group but working individually on separate parts of a group assignment are referred to as working *in a group*. This is not an uncommon situation within an educational setting (Gillies and Boyle, 2011). Cooperation between students might occur, but it is not necessary to accomplish the group’s task. At the end of the task, the students put their separate contributions together into a joint product (Galton and Williamson, 1992; Hammar Chiriac, 2010, 2011a). While no cooperative activities are mandatory while working in a group, cooperative learning may occur. However, the benefits in this case are an effect of social facilitation (Zajonc, 1980; Baron, 1986; Uziel, 2007) and are not caused by cooperation. In this situation, social facilitation alludes to the enhanced motivational effect that the presence of other students have on individual student’s performance.

Working *as a group,* on the other hand, causes learning benefits from collaboration with other group members. Working as a group is often referred to as “real group work” or “meaningful group work,” and denotes group work in which students utilizes the group members’ skills and work together to achieve a common goal. Moreover, working as a group presupposes collaboration, and that all group members will be involved in and working on a common task to produce a joint outcome (Bennet and Dunne, 1992; Galton and Williamson, 1992; Webb and Palincsar, 1996; Hammar Chiriac, 2011a,b). Working as a group is characterized by common effort, the utilization of the group’s competence, and the presence of problem solving and reflection. According to Granström (2006), working as a group is a more uncommon activity in an educational setting. Both approaches might be useful in different parts of group work, depending on the purpose of the group work and type of task assigned to the group (Hammar Chiriac, 2008). Working in a group might lead to cooperative learning, while working as group might facilitate collaborative learning. While there are differences between the real meanings of the concepts, the terms are frequently used interchangeably (Webb and Palincsar, 1996; Hammar Chiriac, 2011a,b; Hammar Chiriac and Granström, 2012).

### PREVIOUS RESEARCH OF STUDENTS’ EXPERIENCES

As mentioned above, there are a limited number of studies concerning the participants’ perspectives on group work. Teachers often have to rely upon spontaneous viewpoints and indications about and students’ experiences of group work in the form of completed course evaluations. However, there are some exceptions (Cantwell and Andrews, 2002; Underwood, 2003; Peterson and Miller, 2004; Hansen, 2006; Hammar Chiriac and Einarsson, 2007; Hammar Chiriac and Granström, 2012). To put this study in a context and provide a rationale for the present research, a selection of studies focusing on pupils’ and/or students’ experiences and conceptions of group work will be briefly discussed below. The pupils’ and/or students inside knowledge group work may present information relevant in all levels of educational systems.

Hansen (2006) conducted a small study with 34 participating students at a business faculty, focusing on the participants’ experiences of group work. In the study different aspects of students’ positive experiences of group work were identified. For example, it was found to be necessary that all group members take part and make an effort to take part in the group work, clear goals are set for the work, role differentiation exists among members, the task has some level of relevance, and there is clear leadership. Even though Hansen’s (2006) study was conducted in higher education, these findings may be relevant in other levels in educational systems.

To gain more knowledge and understand about the essence behind high-quality group work, Hammar Chiriac and Einarsson (2007) turned their focus toward students’ experiences and conceptions of group work in higher education. A primary aim was to give university students a voice in the matter by elucidating their students’ points of view and how the students assess working in groups. Do the students’ appreciate group projects or do they find it boring and even as a waste of time? Would some students prefer to work individually, or even in “the other group?” The study was a part of a larger research project on group work in education and only a small part of the data corpus was analyzed. Different critical aspects were identified as important incitements for whether the group work turned out to be a success or a failure. The students’ positive, as well as negative, experiences of group work include both task-related (e.g., learning, group composition, participants’ contribution, time) and socio-emotional (e.g., affiliation, conflict, group climate) aspects of group work. The students described their own group, as well as other groups, in a realistic way and did not believe that the grass was greener in the other group. The same data corpus is used in this article (see under Section The Previous Analysis). According to Underwood (2003) and Peterson and Miller (2004), the students’ enthusiasm for group work is affected by type of task, as well as the group’s members. One problem that recurred frequently concerned students who did not contribute to the group work, also known as so-called free-riders (Hammar Chiriac and Hempel, 2013). Students are, in general, reluctant to punish free-riders and antipathy toward working in groups is often associated with a previous experience of having free-riders in the group (Peterson and Miller, 2004). To accomplish a favorable attitude toward group work, the advantages of collaborative activities as a means for learning must be elucidated. Furthermore, students must be granted a guarantee that free-riders will not bring the group in an unfavorable light. The free-riders, on the other hand, must be encouraged to participate in the common project.

Hammar Chiriac and Granström (2012) were also interested in students’ experiences and conceptions of high-quality and low-quality group work in school and how students aged 13–16 describe good and bad group work? Hammar Chiriac and Granström (2012) show that the students seem to have a clear conception of what constitutes group work and what does not. According to the students, genuine group work is characterized by collaboration on an assignment given by the teacher. They describe group work as working together with their classmates on a common task. The students are also fully aware that successful group work calls for members with appropriate skills that are focused on the task and for all members take part in the common work. Furthermore, the results disclose what students consider being important requisites for successful versus more futile group work. The students’ inside knowledge about classroom activities ended up in a taxonomy of crucial conditions for high-quality group work. The six conditions were: (a) organization of group work conditions, (b) mode of working in groups, (c) tasks given in group work, (d) reporting group work, (e) assessment of group work, and (f) the role of the teacher in group work. The most essential condition for the students seemed to be group composition and the participants’ responsibilities and contributions. According to the students, a well-organized group consists of approximately three members, which allows the group to not be too heterogeneous. Members should be allotted a reasonable amount of time and be provided with an environment that is not too noisy. Hence, all six aspects are related to the role of the teacher’s leadership since the first five points concern the framework and prerequisites created by the teacher.

Näslund (2013) summarized students’ and researchers’ joint knowledge based on experience and research on in the context of shared perspective for group work. As a result, Näslund noticed a joint apprehension concerning what constitutes “an ideal group work.” Näslund (2013) highlighted the fact that both students and researchers emphasized for ideal group work to occur, the following conditions were important to have: (a) the group work is carried out in supportive context, (b) cooperation occurs, (c) the group work is well-structured, (d) students come prepared and act as working members during the meetings, and (e) group members show respect for each other.

From this brief exposition of a selection of research focusing on students’ views on group work, it is obvious that more systematic studies or documentations on students’ conceptions and experiences of group work within higher education are relevant and desired. The present study, which is a reanalysis of a corpus of data addressing the students’ perspective of group, is a step in that direction.

### AIM OF THE STUDY

The overarching knowledge interest of this study is to enhance the body of knowledge regarding group work in higher education. *The aim* of this article is to add knowledge and understanding of what the essence behind successful group work in higher education is by focusing *on the students’ experiences and conceptions of group work and learning in groups*, an almost non-existing aspect of research on group work until the beginning of the 21st century. A primary aim is to give university students a voice in the matter by elucidating the students’ positive and negative points of view and how the students assess learning when working in groups. Furthermore, the students’ explanations of why some group work results in positive experiences and learning, while in other cases, the result is the opposite, are of interest.

## MATERIALS AND METHODS

To capture university students’ experiences and conceptions of group work, an inductive qualitative approach, which emphasizes content and meaning rather than quantification, was used (Breakwell et al., 2006; Bryman, 2012). The empirical data were collected through a study-specific, semi-structured questionnaire and a qualitative content analysis was performed (Mayring, 2000; Graneheim and Lundman, 2003; Elo and Kyngäs, 2007).

### PARTICIPANTS

All participating students attended traditional university programs where group work was a central and frequently used pedagogical method in the educational design. In addition, the participants’ programs allowed the students to be allocated to the same groups for a longer period of time, in some cases during a whole semester. University programs using specific pedagogical approaches, such as problem-based learning or case method, were not included in this study.

The participants consisted of a total of 210 students, 172 female and 38 male, from two universities in two different cities (approximately division: 75 and 25%). The students came from six different populations in four university programs: (a) The Psychologist Program/Master of Science in Psychology, (b) The Human Resource Management and Work Sciences Program, (c) Social Work Program, and (d) The Bachelor’s Programs in Biology. The informants were studying in their first through eighth terms, but the majority had previous experiences from working in other group settings. Only 2% of the students had just started their first term when the study was conducted, while the vast majority (96%) was participating in university studies in their second to sixth semester.

The teacher most frequently arranged the group composition and only a few students stated that they have had any influence on the group formation. There were, with a few exceptions, between 6 and 10 groups in each of the programs included in this study. The groups consisted of between four to eight members and the differences in sizes were almost proportionally distributed among the research group. The groups were foremost heterogeneous concerning gender, but irrespective of group size, there seems to have been a bias toward more women than men in most of the groups. When there was an underrepresented sex in the group, the minority mostly included two students of the same gender. More than 50% of the students answered that in this particularly group, they worked solely with new group members, i.e., students they had not worked with in previous group work during the program.

### MATERIALS

To collect data about students’ experiences and conceptions of group work, a study-specific, semi-structured questionnaire was constructed. The questionnaire approached the students’ experiences regarding the specific group work they were working in at the time of the data collection (spring 2006), not their experiences of group work in general. The questionnaire contained a total of 18 questions, including both multiple choice and open-ended questions. The multiple choice questions concerned background variables and information about the present group. The seven open-ended questions were designed to gather data about the students’ experiences and perceptions of group work in higher education. The questionnaires were distributed to the different populations of students (some populations studied at the same program) at two universities in Sweden. During the time the questionnaires were completed, the researcher or an assistant was present to answer possible questions. In all, 210 students answered the questionnaire.

### ANALYSIS

#### The previous analysis

As described above (Section Previous Research of Students’ Experiences) a previous analysis based on the same data corpus revealed that most of the students included in the study found group work to be an enjoyable and stimulating working method (Hammar Chiriac and Einarsson, 2007). The data were analyzed using a qualitative content analysis based on three different research questions. There were two main criticisms of the previous study presented from other researchers. The criticism conveyed applied mostly to the question of whether we could assemble these groups into a joint research group and second to the fact that the results were mostly descriptive. To counter this criticism and to elaborate on the analysis, a further analysis was conducted.

#### The present analysis

The present analysis (or reanalysis) was conducted by using an inductive qualitative content analysis based on three open-ended research questions:

(1) In what ways does group work contribute to your learning?

(2) What positive experiences have you had while working in your present group?

(3) What negative experiences have you had while working in your present group?

Each question corresponds to one aspect of the research’s objective, but together, they might support and enrich each other and unravel new information based on the students’ experiences and conceptions of group work. Research question 1, listed above, was not included in the first analysis and is being investigated for the first time in this study, while the other two questions are being reanalyzed. An inductive, qualitative content analysis is applicable when the aim of the research is a description of the meaning or of a phenomenon in conceptual form (Mayring, 2000; Graneheim and Lundman, 2003; Elo and Kyngäs, 2007).

The analysis was carried out over several steps, following the basic principles of an inductive, qualitative content analysis (Mayring, 2000; Graneheim and Lundman, 2003; Elo and Kyngäs, 2007). The steps included three phases: preparation, organizing, and reporting (Elo and Kyngäs, 2007). Each question was treated as a unit of analysis and was thus analyzed separately. In the *preparation* phase, the researcher tried to make sense of the data by becoming familiar with the data corpus. In the current study, this included transcription and thorough reading of the answers. An open coding system composed of marginal notes and headings began the second phase, which included *organizing* the data. This second phase, in turn, included open coding, creating categories, and abstraction. The notes and the headings from the open coding were transferred to coding sheets and then grouped into categories. Categories were formed through the interpretation of the codes that described the same meaning or phenomenon. Finally, an abstraction process began, where a general description of the grouped categories formed an abstraction (see **Table 1**). An abstraction was denominated using the content-characteristic words for this paper: *learning, study-social function,* and *organization*. The third phase, *reporting*, addressed the presentation of the process of analysis and the results.

**Table 1:** Examples from the organization phase of the coding process.

Abstractions	Categories	Codes (examples)
Learning	Facilitate
	- Academic learning	- Learn more
		- Discussing and questioning
		- New perspectives
	- “Group knowledge”	- Learn about groups by working in groups
		- Social training
		- Interpersonal skills
	Hamper learning	- Out of focus
		- Ineffective
		- Conflicts

Study-social function	Facilitate
	- Affiliation	- Membership
		- Belonging
		- Friends
	- For the individual student	- Relief
		- Support
		- Motivation
		- Confirmation
	Hamper	- Group climate
		- Negative conceptions
		- Influenced by bad temper

Organization	Facilitate	- Group composition
		- Group structure
		- Way of working
		- Contributions
	Hamper	- Group composition
		- Group structure
		- Way of working
		- Contributions

The final aim of this study is to present the phenomenon studied in a model or conceptual map of the categories (Elo and Kyngäs, 2007). In following these procedures, we aim to expand our understanding of the existing work and to counter the second part of the criticisms, which included criticisms stating that the results were mostly descriptive in nature. To counter the criticisms regarding the question of whether we could assemble these groups into a joint research group, the qualitative abstraction that emerged from the qualitative content analysis was compared to background information by using SPSS. Three background variables were used: gender, cities, and programs.

### ETHICS AND QUALITY

The ethical principles provided by the British Psychology Society have formed a guideline [British Psychology Society (BPS), 2006] for the present study. The ethical principles, which emphasize the concern for participants’ interest, have been applied throughout the study [American Psychological Association (APA), 2002; British Psychology Society (BPS), 2004; Barett, 2007]. To facilitate trustworthiness, a thorough description of the analysis process has been presented (Graneheim and Lundman, 2003; Elo and Kyngäs, 2007). Translated citations are also included to increase trustworthiness.

## RESULTS

As described above, the analysis resulted in three abstraction emerging: *learning, study-social function*, and *organization*. Each abstraction includes both a positive variant (i.e., facilitating learning, study-social function, and/or organization) as well as a negative alternative (i.e., hampering learning, study-social function, and/or organization). The results will be presented in three different sections, with each section corresponding to one abstraction. However, we would like to call attention to the fact that one fifth (20%, including missing value 8%) of the students included in this study did not perceive and/or mention any negative experiences at all in their present group. From a general point of view, there is no difference with respect to gender or city regarding the distribution of positive and negative experiences concerning the abstractions, neither concerning different programs nor the distribution of negative experiences (all *p* > 0.05). In contrast, there is a difference between the various programs and the distribution of positive experiences (χ^2^ = 14.474; df: 6; *p* < 0.025). The students from the social work program display a higher amount of positive experiences in connection with a study-social function and organizing in comparison with the other programs.

### LEARNING

The majority of the students (97%) responded that working in group somehow *facilitated learning,* academic knowledge, collaborative abilities or both. They learned more or different things when working in groups than they would have if working alone. By discussing and questioning each other’s points of view and listening to their fellow students’ contributions, thus obtaining different perspectives, the participants experienced an enhanced academic learning, compared to working alone. “I learn much more by working in groups than working individually. I obtain more through interaction with the other group members.” Academic knowledge is not the only type of knowledge learned through group work. In addition to academic knowledge, students also gain advanced knowledge about how groups work, how the students function as individual members of groups and how other members behave and work in groups. Some of the respondents also argued that group work in group courses strengthen the combination between empirical and theoretical learning, thus learning about groups by working in groups. “Through practical knowledge demonstrate several of the phenomena we read about in theory (group psychology and sociology).”

The results show no difference when considering either gender or city. However, when comparing the four programs included in the study and the types of learning, a difference occurs (χ^2^ = 14.474; df: 6; *p* < 0.025). A division into two parts seems to generate the difference. On the one hand, the students from the Bachelor’s Program in Biology and the students from the Human Resource Management and Work Sciences Program emphasize academic knowledge. On the other hand, students from the Psychologist Program/Master of Science in Psychology and Social Work Program more often mentioned learning collaborative abilities single handed, as well as a combination of academic knowledge and group learning.

Even though the participants did not expressly report that group work *hampered learning,* they often mentioned that they perceived group work as being ineffective due to loss of focus and the presence of conflicts, thereby hampering conceivable learning. One respondent stated, “that you sometimes are out of focus in the discussion and get side-tracked instead of considering the task.” Another offered the following perspective: “Occasionally, it is too little task related and feels unnecessary sometimes. Individual work is, in certain situations, preferable.” Group work might be perceived as ineffective and time consuming considering long working periods with tedious discussions. One participant stated, “The time aspect, everything is time consuming.” The absence or presence of conflicts in the group affects students’ experiences, and conflicts not handled may influence learning in a negative way. The students perceived that it was difficult to come to an agreement and experience those conflicts and the need to compromise hampered individual learning. Accordingly, the absence of conflicts seemed to be an important incitement for learning. However, fear of conflicts can lead to reduced learning and cause negative experiences, but to a considerably lesser extent than does the presence of actual conflicts. “A great fear of conflicts sometimes raises an oppressive atmosphere.” “Fear of conflicts leads to much not made known.”

### A STUDY-SOCIAL FUNCTION

Group work also has an important *study*-*social function* according to the students. They describe their membership in groups as an important aspect of affiliation. In general, the total number of students at a program is approximately 60–80 or more. In contexts with a large population of students, the smaller group gives the participants an opportunity to feel affiliated with the group and to each other. “Feels safe to have a certain group to prepare oneself together with before, for instance, an upcoming seminar.” The group gives the individual student a platform of belonging, which might serve as an important arena for learning (*facilitate*) and finding friends to spend leisure time with. Many of the participants also reported feeling a positive atmosphere in the group, which is important for the satisfaction of being in the group together with the fellow students.

To be a member of a group may also serve as a function of relief, both academically and socially, for the individual student. The participants reported that many of the tasks assigned by the university teachers are difficult to handle on their own. “The others explain to me. We help one another.” However, the students reported that they helped and supported each other, even if the task did not demand cooperation. “As a student, you get more active. You help one another to extract the groups’ common knowledge. Forward info if somebody is missing.” Being a member of a group also affects students’ motivation to study. They prepare themselves by reading texts and other material before the next group session. Group work may also have positive effects on achievement. Students’ total amount of time and effort on their work may also increase. Through group work, the participants also get confirmation of who they are and what their capacities are.

Being a member of a group also has its downside, which often has to do with the group climate and/or group processes, both of which have multiple and complex features. Many students reported that both the group climate and group processes might be the source of negative conceptions of the group and *hamper* learning. “Process losses.” The respondents described negative conceptions based on the feeling of not having enough time to get to know each other in the group or being in situations where no cooperation occurred. Other students referred to the fact that the group’s life is too long, which may lead to group members not only wearing each other out, but also having a negative effect on each other’s mood. “Influenced by each other’s mood.” Examples of negative experiences are process losses in general, including insufficient communication, unclear roles, and problems with one group member. As mentioned above, the students from the Social Work Program display a higher number of positive experiences in connection with a study-social function and organizing in comparison with students from the other programs.

### ORGANIZATION

O*rganization* concerns the structure of group work and includes different aspects, all describing group work from different angles. The aspects are relevant no matter how the participants perceive the group work, whether as positive or negative. Unlike the other two abstractions (learning and study-social function), organization includes the same aspects no matter what the experiences are, namely *group composition*, *group structure*, *way of working* and* contributions.*

Whether the group is *composed* in a homogeneous or heterogeneous way seems to be experienced in both a positive and negative sense. A well-thought-out *group composition*, including both group size and mix of members, is essential. A just large-enough group for the task, consisting of a population of members that is not too heterogeneous, *facilitates* a joyful experience and learning. A homogeneous mix of members might be perceived as positive, as the students feel that they have similar life situations, opinions, and skills, thereby causing positive conditions for collaboration within the group. Conversely, in a group with a heterogeneous mix, different members contribute with different knowledge and/or prior experiences, which can be used in the group for collective and collaborative learning. “Good group composition, distribution of age groups that leads to fruitful discussions.”

An additional facilitating prerequisite is that the group develops adequate *ways of working* together, which includes a well-organized *group structure*. Well-working groups are characterized as having developed adequate ways of working together, while groups that work less well together lack a developed way of cooperation. “Well-organized working group with clear and distinct rules and structure.” Preparation and attendance for group work are aspects mentioned as facilitating (and hampering) incitements. Group work in educational settings sometimes entails that you, as a student, are forced to read and learn within a certain period of time that is beyond your control. Some participants find the pressure positive, hence “increase the pressure to read chapters in time.” The members’ *contribution* to the group is also a central factor for the students’ apprehension of how the group works. This is, in short, about how much each member ought to contribute to the group and to the work. Groups considered to be well-working are ones where all members contribute to the group’s work, but the content of the contribution may vary according to the single member’s qualifications. “We work well together (most of us). Everybody participates in different ways and seems committed.” “Good, everybody participates the same amount. We complement each other well.”

The same prerequisites can lead to the reverse result, i.e., *hampering* learning and stirring up negative experiences. If the group members are too identical (a homogeneous *group composition*), it might lead to a lack of opinions, which several participants perceived as being negative. “That we do not get a male perspective about the subject. We are all girls, at the age of 20, which also means that we have pretty much the same experiences that may be seen as both positive and negative. The negative is the lack of opinion.” If the group is considered to be too small, students seems to find it troublesome, as the relationships are few, but there are also few people who are available to handle the workload allotted to the group. Nevertheless, a group that is too large could also lead to negative experiences. “It is far too large a group.”

A lack of *group structure* might lead to a lower degree of satisfaction with the group’s *way of working*. A commonly expressed point of view seen in the students’ answers involved the occurrences of when all members did not attend the meetings (absence). In these cases, it was also viewed that the work in the group often was characterized as unstructured. “Sometimes a bit unclear structures, some students have difficulties with coming in time.” Not attending or coming unprepared or badly prepared to the group work is other aspect that is commented on. “Low degree of fellowship, punctuality is a problem, an insecure group.” Some students find it frustrating to prepare for a certain time decided that is beyond their control. “A necessity to read certain chapters within a specific period of time is never stimulating.”

One characteristic of groups that are not working well is that *contribution* varies among the members. In group work, students with different levels of ambition are assembled, which may result in different levels of interest and commitment, as well as differences in the willingness to take on responsibilities or part of the workload of the group’s work. Some members are active and do much of the work, while others barely contribute at all. “Some don’t do anything while others pull the heaviest burden. Two out of three prepare before the meeting, the rest think that they are able to read during the group work and do not supply the group with anything else other than delays and frustration.” A common answer seen in the questionnaires that concerns negative experiences of group work as they relate to contribution is: “Everybody does not contribute just as much.” or “There is always someone who just glides along and doesn’t take part.”

### SUMMARY OF THE RESULTS

**FIGURE 1:**
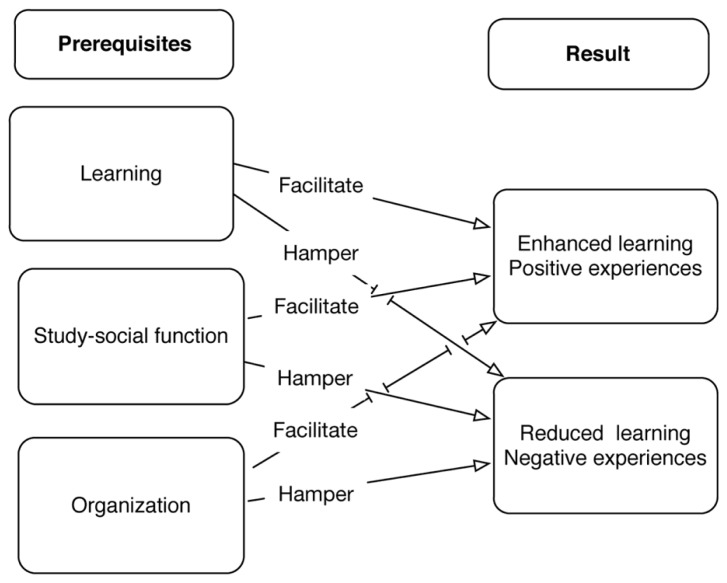
**A model illustrating the relationship between abstractions and result**.

The results are summarized in a model illustrating the relationship between abstractions (i.e., learning, study-social function, and organization) and result (i.e., enhanced or reduced learning), as well as positive or negative experiences (see **Figure 1**).

**FIGURE 2:**
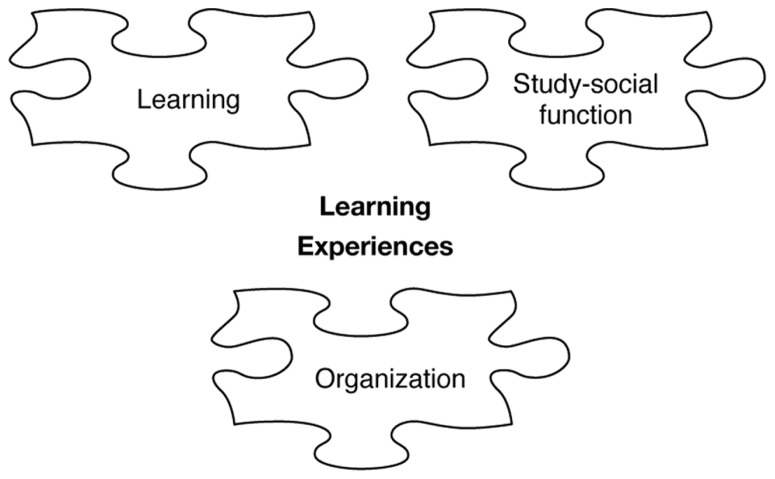
**Pieces of jigsaw puzzle influence learning and experiences**.

The figure shows that all three abstractions may facilitate or hamper learning as well as the experiences of group work. To piece together, the difficult and extensive jigsaw puzzle concerning why some group work result in positive experiences and learning, while in other cases the result is the reverse is still not solved. In this article, we propose that the prerequisites learning, study-social function, and organization influence learning and experiences of working in group, thus, providing additional pieces of information to the jigsaw puzzle (**Figure 2**).

## DISCUSSION

The current study focuses on university students’ experiences and conceptions of group work and learning in groups. A primary aim was to give university students a voice in the matter by elucidating the students’ positive and negative points of view, as well as how the students’ assess learning when working in groups. The analysis resulted in the emergence of three different abstractions: learning, study-social function, and organizations. Each abstraction also included a positive and a negative variant. In other words, all three abstractions either facilitated or hampered university students’ learning, as well as their experiences of group work.

### LEARNING IN GROUP WORK

The result shows that the majority of the students (97%) experience that working in group *facilitated learning,* either academic knowledge, collaborative abilities or both, accordingly confirming previous research (Johnson and Johnson, 2004; Baines et al., 2007; Gillies and Boyle, 2010, 2011). According to the students, they learn more or different things when working in groups compared with working individually. Academic knowledge was not the only type of knowledge learned through group work. In addition to academic knowledge, students also gained advanced knowledge about how groups work, how the students function as individual members of groups and how other members behave and work in groups. Some of the respondents also argued that group work might strengthen the combination between empirical and theoretical learning, thus the students were learning about groups by working in groups. This implies that group work, from a learning perspective, serves several functions for the students (Kutnick and Beredondini, 2009; Gillies and Boyle, 2010, 2011; Hammar Chiriac, 2011a,b). Group work also seems to have an important study-social function for the university students, hence confirming that group work serves more functions than just being a pedagogical mode.

Affiliation, fellowship, and welfare seem to be highly important, and may even be essential prerequisites for learning. Accordingly, group work functions as both as an objective (i.e., learning collaborative abilities), and as the means (i.e., a base for academic achievement), or both, for the students (Gillies, 2003a,b; Johnson and Johnson, 2004; Baines et al., 2007). Moreover, the students from the Bachelor’s Program in Biology and the students from the Program for Human Resources seem to use group work more as means for obtaining academic knowledge. In contrast, students from the Psychologist Program/Master of Science in Psychology and Social Work Program more often mentioned learning collaborative abilities alone, as well as a combination of academic knowledge and group learning, thus using group work as an objective, as a means, or as a combination of both. One interpretation might be that the type of task assigned to the students differs in various programs. This can be valid both concerning the purpose of group work (group work as objective or as the means), but also arrangement (working in a group or as a group; Underwood, 2003; Hammar Chiriac and Granström, 2012). Another possible explanation might be that the main emphasis in the Bachelor’s Program in Biology and the Program for Human Resources is on product and academic knowledge, while in the Psychologist Program/Master of Science in Psychology and Social Work Program, the process is more articulated and demanded. However, this is only speculation and further research is needed.

Even though the participants did not explicitly state that group work *hampered learning,* they mentioned that they perceived group work to be ineffective due to the loss of focus and/or the presence of conflicts with other group members, thereby hampering conceivable learning. This may also be an effect of the purpose or arrangement of the group work (Cantwell and Andrews, 2002; Underwood, 2003; Peterson and Miller, 2004; Hansen, 2006; Hammar Chiriac and Granström, 2012; Hammar Chiriac and Hempel, 2013).

### EXPERIENCES OF GROUP WORK

The results revealed that several aspects of group work are important incentives for learning. In addition, this study revealed students’ *experiences of group work* (i.e., facilitating or hampering positive/negative experiences), which is in line with the previous studies on students’ experiences of working in groups (Cantwell and Andrews, 2002; Underwood, 2003; Peterson and Miller, 2004; Hansen, 2006; Hammar Chiriac and Granström, 2012; Hammar Chiriac and Hempel, 2013). Group composition, group structure, ways of working, and participants’ contributions are aspects put forward by the university students as either facilitating or hampering the positive experience of group work (Underwood, 2003; Peterson and Miller, 2004; Hansen, 2006; Hammar Chiriac and Granström, 2012; Hammar Chiriac and Hempel, 2013).

Several of the aspects bear reference to whether the group members work *in a group* or* as a group* (Underwood, 2003; Hammar Chiriac and Granström, 2012). Working as a group is characterized by common effort, utilization of the group’s competence, and includes problem solving and reflection. All group members are involved in and working on a common task to produce a joint outcome (Bennet and Dunne, 1992; Galton and Williamson, 1992; Webb and Palincsar, 1996; Hammar Chiriac, 2011a,b). According to the results, not all groups are working as a group but rather working in a group, which, according to Granström (2006), is common in an educational setting.

Due to problems with group composition, members’ contributions, and group structure, including rules and ways of cooperation, some students end up with negative experiences of group work. Additionally, the university students allude to the fact that a well-functioning supportive study-social context is an essential prerequisite not only for positive experiences of group work, but also for learning (Hammar Chiriac and Hempel, 2013). Both working in a group and working as group might be useful in different parts of the group work (Hammar Chiriac, 2008) and cause learning. Hence working in a group causes cooperative learning based on social facilitation (Zajonc, 1980; Baron, 1986; Uziel, 2007) while working as group causes learning benefits through collaboration with other group members. Although both approaches might cause positive or negative experiences, a conceivable interpretation is that working as a group has a greater potential to enhance positive experiences. The findings suggest a need for further research to fully understand why some group work causes positive experiences and other instances of group work cause negative experiences.

The findings in the current study develop the findings from Hammar Chiriac and Einarsson (2007). First, it shows that it is possible to assemble all groups in to a joint research group (see below). Second, a thorough reanalysis, using an inductive qualitative content analysis, resulted in the emergence of three different abstractions: learning, study-social function, and organizations as either facilitating or hampering learning, and experiences.

### METHODOLOGICAL CONSIDERATIONS

There are some limitations in the current study and most of them have to do with the construction of the study-specific, semi-structured questionnaire. First, the questions do not discriminate between (a) the type of group work, (b) the purpose with the group work, (c) the structure of the group work (i.e., extent and/or time); or (d) ways of working in the group (i.e., cooperation or collaboration). Second, the design of the questionnaire does not facilitate comparison between the populations included in the group. The questionnaire treated group work as one activity and did not acknowledge that group work can serve different functions and include various activities (Hammar Chiriac, 2008). This simplification of the phenomena group work causes criticism concerning whether or not it is possible to assemble these populations into a joint research group. An elaborated description of the analysis process and the comparison to three background variables has been used to counter this criticism. The thin results from the comparison, indicate that based on the question used in the study-specific questionnaire, it is possible to assemble the results into a corpus of joint results.

## CONCLUSION/CONCLUDING REMARKS

The results indicate that most of the students’ experienced that group work facilitated learning, especially concerning academic knowledge. Three important prerequisites (learning, study-social function, and organization) for group work that serve as an effective pedagogy and as an incentive for learning were identified and discussed. All three abstractions either facilitated or hampered university students’ learning, as well as their experiences of group work. By listening to the university students’ voices and elucidating their experiences and conceptions, we have been able to add new knowledge and understanding of what the essence is behind successful group work in higher education. Furthermore, the students’ explanations of why some group work results in positive experiences and learning, while in other cases, the result is the opposite, can be of use for further development of group work as a pedagogical practice.

## Conflict of Interest Statement

The author declares that the research was conducted in the absence of any commercial or financial relationships that could be construed as a potential conflict of interest.
